# Co-administration of isoprenaline and phenylephrine induced a new HFrEF mouse model through activation of both SNS and RAAS

**DOI:** 10.3389/fcvm.2025.1531509

**Published:** 2025-03-10

**Authors:** Huimin Su, Ming Liu, Siteng Wang, Beiduo Tian, Hao Hu, Li-Kun Ma, Jianyuan Pan

**Affiliations:** ^1^ Department of Cardiology, The First Affiliated Hospital of USTC, Division of Life Sciences and Medicine, University of Science and Technology of China, Hefei, China; ^2^ Department of Cardiology, The First Hospital of Xinjiang Medical University, Urumqi, China; ^3^ Department of Cardiology, The Second People’s Hospital of Hefei, Hefei Hospital Affiliated to Anhui Medical University, Hefei, Anhui, China

**Keywords:** heart failure, drug modelling, mouse model, SNS, RAAS

## Abstract

**Introduction:**

The pathogenesis of human heart failure is diverse, and a large number of animal models have emerged to better understand the development of heart failure in humans. Among them, there are several methods of induction in mouse heart failure models, each with its advantages and disadvantages. The use of drug induced heart failure models has greatly facilitated basic research and reduced the disadvantages of time-consuming and labor-intensive surgical modeling.

**Methods:**

In our experiments, we used a combination of isoprenaline (ISO) and phenylephrine (PE) for modeling; we aimed to evaluate whether it is superior to conventional drug-induced models, especially those induced by isoprenaline alone. The ISO and PE were administered for 2 weeks by subcutaneous implantation with a micro-osmolar pump, and the mice were monitored dynamically for cardiac ultrasound and blood pressure.

**Results:**

RNA sequencing of myocardial tissues after execution of mice further clarified that hypertrophy, fibrosis genes, Sympathetic nervous system (SNS), and Renin-angiotensin-aldosterone system (RAAS) pathways were upregulated.

**Discussion:**

Therefore, we conclude that the ISO/PE-induced mouse heart failure model can activate both the SNS and RAAS, through the activation of both α-adrenergic receptor (α-AR) and β-adrenergic receptor (β-AR), which is more consistent with the development of human heart failure than the ISO-induced model and is expected to be a unique and representative heart failure modeling method.

## 1 Introduction

Heart failure (HF) is the end-stage of many cardiovascular diseases, affecting the quality of life of 1%–2% of the global population, approximately 40 million people (1). The sympathetic nervous system (SNS), the renin-angiotensin-aldosterone system (RAAS), and the natriuretic peptide system (NPS) were the three key roles in the development of heart failure (2). The natriuretic peptide system displays a protective role in the progression of heart failure through water/sodium drainage, vasodilation, and inhibition of aldosterone secretion (3). The SNS and RAAS were activated earlier during the development of heart failure, although in the short term they can play a compensatory role by enhancing myocardial contractility, promoting water and sodium retention, and constricting peripheral vasculature (4), long-term activation of both would lead to deterioration of heart function through cardiac hypertrophy and fibrosis (5). For this reason, β-blockers, angiotensin-converting enzyme inhibitors (ACEI), and aldosterone receptor antagonists have been used clinically to counteract the prolonged activation of both systems and can benefit patients with heart failure (4, 6, 7).

In the past, HF mouse model constructions include two types; the first type was drug-induced HF model such as isoprenaline, angiotensin II, doxorubicin, etc. (8–10); the second one was surgical modeling such as aortic arch constriction (TAC) and anterior descending coronary artery ligation (11, 12). The degree of TAC modeling narrowing is not easily controlled and requires high operator competence (13), anterior descending branch ligation is more widely used in the field of ischemia-reperfusion studies (14). Therefore, many basic researchers prefer to use drug-induced HF models due to their convenience and stability. Isoprenaline, a non-selective β-receptor agonist, acts mainly through activation of the sympathetic nervous system (15); previous studies found that the ISO-induced HF mouse model mainly shows the upregulation of cardiac fibrosis and inflammatory gene expression (16) due to the less activation of RAAS. Angiotensin II, on the other hand, mainly induced cardiac hypertrophy rather than cardiac fibrosis through activation of RAAS, has little effect on the sympathetic nervous system, and could not fully simulate human HF development (17). Doxorubicin-induced heart failure is mainly achieved by destroying cardiomyocytes, and the predominantly apoptotic changes are not consistent with the progression of heart failure in humans (18). Until now, no animal model of heart failure has been able to fully simulate the progression and development of human heart failure through both activations of RAAS and SNS.

In the development of HF, it is important to note that sympathetic nervous system activation can occur not only through β-adrenergic receptors (β-ARs) but also by agonizing α-adrenergic receptors (α-ARs) (19). There are 2 principal types of α-ARs, α1-AR, and α2-AR, α1-ARs are the classic postsynaptic α receptors and are widely expressed in vascular smooth muscle, which participates in blood pressure control (20). Recent studies have found that ISO promotes the progression of heart failure by inducing MD2 activation in cardiomyocytes via the β1-AR-cAMP-PKA-ROS signalling axis and inflammatory responses in macrophages via the β2-AR-cAMP-PKA-ROS axis.

The expression of the cardiac α1-AR changes during the course of heart failure. Normally, the percentage of α1-AR in the heart is about 10%, whereas in heart failure, its expression is upregulated to about 25%. This increase in expression is associated with the pathophysiological mechanisms of heart failure, and α1-AR plays important adaptive roles in heart failure, including enhancing myocardial contractility, modulating myocardial adaptive hypertrophy, preventing cell death, and protecting against ischaemic injury. Thus, α1-AR plays a complex role in the process of myocardial hypertrophy, both as part of adaptive remodelling of the heart and potentially contributing to the deterioration of cardiac function in long-term pathological states. Investigating the mechanism of action of α1-AR is important for understanding the pathophysiological process of cardiac hypertrophy and the development of therapeutic strategies. α1-AR activation of phospholipase C β1 at the nuclear membrane leading to myo-inositol [1,4,5]-triphosphate-dependent nuclear release of Ca^2+^ histone deacetylase 5 (HDAC5) and CAMKII-induced nuclear export are central mechanisms for the induction of heart failure (21). The activation of phospholipase C β1 at the nuclear membrane by α1-AR, leading to the release of Ca^2+^ histone deacetylase 5 (HDAC5) and CAMKII-induced nuclear export, is a central mechanism in the induction of heart failure (22). In our study, we co-administrated ISO and PE via minipump to chronic perfusion and induced a novel mouse HF model which could activate both β-ARs and α1-ARs (23) to mimic the stimulation of RAAS and SNS. Our experimental results demonstrated that the combined use of ISO and PE-induced mouse model could better simulate the developmental process of human HF through the activation of RAAS and SNS. In addition to an early onset of heart failure phenotype, ISO/PE group can better induce cardiac hypertrophy and cardiac fibrosis; the upregulation of related genes was also more obvious than in the ISO-induced mouse model.

## 2 Materials and methods

### 2.1 Animals

All animal care and experimental procedures were approved by the Animal Policy and Welfare Committee of the First Affiliated Hospital of the University of Science and Technology of China (Approval File No. AF/SC-12-2/04.0). All animals received humane care according to NIH guidelines (Guidelines for the Care and Use of Laboratory Animals). The animals used for the experimental modeling were 6–8 weeks old wild-type C57BL/6JGpt male mice, purchased from Collective Pharmachem (Nanjing, China). The mice were raised in the animal house of Hefei University of Technology. The living environment of the mice was maintained at room temperature of 24 ± 1°C and humidity of 50%±10%; day and night hours were 12 h (light was provided from 8:00 a.m. to 8:00 p.m.); and they were fed and watered extensively.

### 2.2 ISO/PE micro-osmosis pump subcutaneous implantation

Mice were acclimatized to feeding for one week and then subjected to micro-osmotic pump implantation modeling. The dosage of ISO (SIGMA, #BCCG5557) and PE (Tocris, #BP284) were both 30 mg/kg/d24. The average volume of the osmotic pump was 246.6 ul (alzet, #10135-05). The pump was dispensed one day before micro-osmotic pump implantation, placed in saline and stored overnight at 37°C protected from light; the mice were depilated using hair removal cream, prepared for the skin. Isoflurane was used to induce and maintain anesthesia, and the dorsal skin was incised, the skin and subcutaneous tissue were separated, incorporated into a micro-osmotic pump, and sutured. After implantation, the mice were placed on a heating pad and observed for 10 min to determine normal vital signs before being placed in the cage for further feeding. In this experiment, the mice were divided into three groups, the control group only had saline (0.9% NaCl solvent) in the osmotic pump, and the other two experimental groups had ISO and ISO/PE in the osmotic pump, respectively. According to previous reports in the literature, ISO-induced heart failure was modelled for 2 weeks. Therefore, the duration of modelling in this study was determined to be 2 weeks.

### 2.3 Transthoracic echocardiography and blood pressure monitoring

Ultrasound and blood pressure monitoring were performed before and 3, 7, and 14 days after modeling, respectively. The mice were depilated with hair removal cream before ultrasound; ultrasound measurements were performed after isoflurane induction and maintenance anesthesia. During the image acquisition process, we closely monitored the heart rate and respiration of the mice, and adjusted the concentration of anesthetics as needed, while dopamine was given to maintain the heart rate within 400–500 bpm, and mimic heart failure and its movement during exercise condition. Ultrasound measurements were taken in both the transverse and longitudinal axes to dynamically observe changes in cardiac function, with left ventricular ejection fraction and ventricular wall thickness as the main indicators. Blood pressure measurements were performed in the animal room, and the systolic and diastolic blood pressures and heart rates were measured 5 min after adaptation. In the quiet state, we tested the blood pressure of mice in the awake state. We attached a sensor to the tail of the mouse and monitored the blood flow signal by inflating and deflating the caudal artery while simultaneously applying and releasing pressure to derive the blood pressure value. Ultrasound equipment was purchased from VINNO (VINNO, Suzhou, China), and the blood pressure instrument was purchased from Zongshi Technology Company (Zongshi, Beijing, China).

### 2.4 Hematoxylin-eosin (HE) staining and masson staining

Heart tissues were excised from mice after execution, preserved in 4% paraformaldehyde, dehydrated in a dehydrator, embedded in paraffin, sectioned, and stained with HE and Masson. Masson staining was performed using Masson trichrome staining solution (Baso, #BA4079A) purchased from Baso, HE staining was performed using hematoxylin staining solution from biosharp, and eosin solution was eosin powder purchased from SenBeiJia, which was prepared ready to use.

### 2.5 RT-qPCR

Ventricular tissues were excised, added to Trizol, and placed in negative 80°C for freezing, tissue RNA was extracted, reverse transcribed, and subjected to RT-qPCR and experimental data were analyzed using LightCycler® 96 SW 1.1 software. The primer sequences used were shown in Table 1.

**Table 1:** Primer sequences for RT-qPCR.

Gene	Species	Primer sequences
Nppb	Mus musculus	Forward: CAGTCTCCAGAGCAATTCAAGATG
		Reverse: ACAACAACTTCAGTGCGTTACA
Myh7	Mus musculus	Forward: AGGTCTGGCTCTGAGCATTC
		Reverse: CCTTTCTCGGAGCCACCTTG
Col1a1	Mus musculus	Forward: CGATGGATTCCCGTTCGAGT
		Reverse: GAGGCCTCGGTGGACATTAG
Postn	Mus musculus	Forward: AAGGCGAAACGGTGACAGAA
		Reverse: ACAACAACTTCAGTGCGTTACA
IL-6	Mus musculus	Forward: GACAAAGCCAGAGTCCTTCAGA
		Reverse: TGTGACTCCAGCTTATCTCTTGG
Ppia	Mus musculus	Forward: TCAACCCCACCGTGTTCTTC
		Reverse: CCAGTGCTCAGAGCTCGAAA

### 2.6 Western blotting

After the mice were executed, the ventricular muscle tissue was excised and frozen at –80°C. Lysis was added with protease inhibitors and phosphatase inhibitors for lysing the tissues to extract proteins. Protein concentration was determined by the Bradford assay (item 5000205, Bio-Rad, California, USA). Samples were separated using sodium dodecyl sulfate-polyacrylamide gel electrophoresis (SDS-PAGE) and electrotransferred to a PVDF membrane. The membranes were then closed in 5% skimmed milk for 1 h at room temperature. Primary antibodies were incubated at 4°C overnight. The membrane is then incubated with the appropriate HRP coupled secondary antibody for 1 h at room temperature. According to previous studies, the main members mediating cardiac remodeling are the mitogen-activated protein kinase (MAPK) family (24). Therefore, our experiments focused on monitoring the phosphorylation levels of its main members, ERK, JNK, and P38, to assess their activation status. The internal reference used in the experiment is Vinculin (1:1,000, CST, #13901). Other antibodies used in the experiment include ERK1/2(1:1,000, CST, #4695), Phospho-ERK1/2 (Thr202/Tyr204)(1:1,000, Proteintech, 80031-1-RR), JNK(1:10,000, Proteintech, 66210-1-Ig), Phospho-JNK(Try185)(1:2,000, Proteintech, 80024-1-RR), p38(1:1,000, CST. #8690), Phospho-p38 (Thr180/Tyr182)(1:1,000, CST, #4511). Secondary antibody using Goat-anti-Rabbit (1:10,000, LI-COR, 926-68071) or Goat-anti-Mouse (1:10,000, LI-COR, 926-32210) fluorescent secondary antibody. Stripes were imaged with the Odyssey® DLx imaging system (LI-COR Odyssey, DLx) in the 800 nm or 700 nm channel. Quantify the band density using ImageJ analysis software.

### 2.7 RNA sequencing

Tissues from the right and left ventricles of mice were excised after execution and placed in RNA seq preservation solution, and sent to Lianchuan Biotech (Hangzhou, China) for sequencing. The preservation solution used for sequencing was provided by the company, and the data were processed and mapped using the Unichuan BioCloud platform (OmicStudio) and Rstudio. At the same time, the sequencing results were further validated by enrichment analysis of the Kyoto Encyclopedia of Genes and Genomes (KEGG) using the platform database.

### 2.8 Experimental design

**Figure 1:**
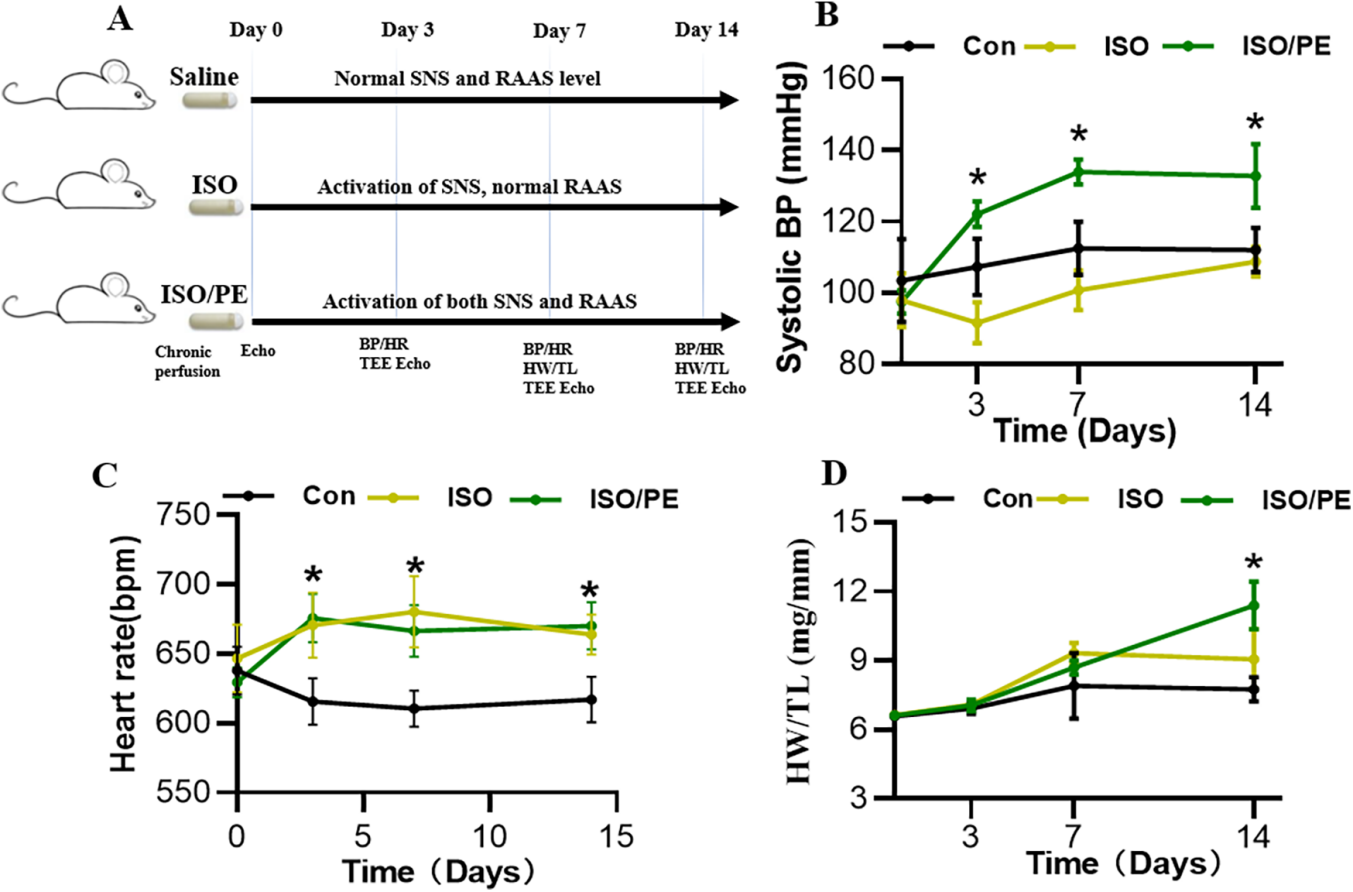
Experimental design and a comparation among 3 groups in terms of systolic BP, heart rate and HW/TL. **(A)** Experimental design: mouse model was constructed using subcutaneous implantation of micro-osmotic pump, divided into control group, ISO (30 mg/kg/day) group and ISO/PE (30 mg/kg/day) group; 6 mice in each group. Cardiac ultrasound monitoring and blood pressure monitoring were performed before and 3, 7 and 14 days after surgery, respectively; mice were executed at 1 and 2 weeks, respectively, and the heart-to-tibial length (HW/TL) ratio was measured. **(B)** Systolic pressure and **(C)** heart rate both monitored by blood pressure monitors through the tail root. **(D)** HW/TL ratio was obtained by measuring heart weight and tibia length after execution of mice on days 0, 3, 7, and 14 of modeling. Individual data, mean ± SEM of *n* = 6 animals are shown. *P* values were determined by repeated measures one-way ANOVA. *P < 0.05.

In this experiment, a mouse model was constructed using subcutaneous implantation of a micro-osmotic pump, divided into a control group, ISO group, and ISO/PE group; 6 mice in each group. The ISO-induced mouse model has been widely used, and the dosage of ISO and PE were taken as 30 mg/kg/day in this experiment with reference to the former approach of modeling (15). The experimental design is shown in Figure 1A. One week after the mice were acclimated to rearing, the anterior thoracic region was depilated and preoperative ultrasound monitoring was performed. Afterward, the back is depilated and the micro-osmosis pump is embedded under the skin. Isoflurane was used for induction and maintenance of anesthesia for both ultrasound and micro-osmotic pump implantation. To better compare the differences and effects between the ISO group and ISO/PE group, we gave cardiac ultrasound and blood pressure monitoring before and 3, 7, and 14 days after modeling, respectively. Cardiac ultrasound mainly monitors ejection fraction (EF) and fractional shortening (FS) and other cardiac function-related indicators.

### 2.9 Statistical analysis

Statistical analyses were performed with GraphPad Prism 8. The results of data were presented uniformly using mean ± SEM, and an unpaired t-test was used for the comparison between two groups; one-way ANOVA was used for the comparison among three groups. GraphPad Prism 8 was used for graphing; Image J was used for staining and western blot quantitative analysis. *P* < 0.05 was considered statistically significant.

## 3 Results

### 3.1 Characteristics of HFrEF mouse model development and echocardiographic measurements

**Figure 2:**
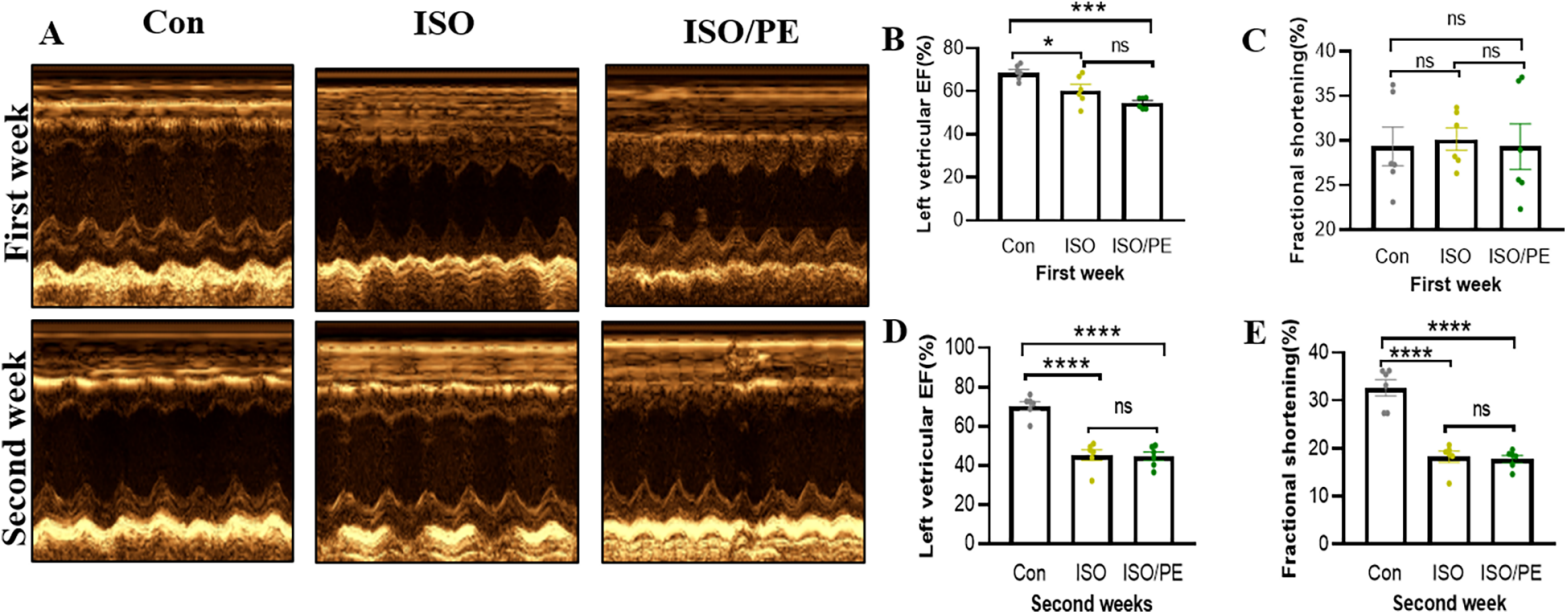
Echocardiographic evaluation of left ventricle EF and FS. **(A)** Echocardiography analysis of cardiac function in control (NaCl), isoprenaline (ISO) and isoprenaline/phenylephrine (ISO/PE) treated mice. Showing ultrasound results after 1 week and 2 weeks of dosing. **(B–E)** Left ventricular ejection fraction (LVEF) and short-axis fractional shortening (% FS) results, statistical analysis was performed at 1 week and 2 weeks after drug administration as monitoring points. Individual data, mean ± SEM of *n* = 6 animals are shown. *P* values were determined by repeated measures one-way ANOVA. *P < 0.05, ***P < 0.001, ****P < 0.0001.

The survival rate of mice after the operation was 100%, no infection appeared, and the mobility, feeding condition, and mental status were comparable to the control group. Dynamic mouse tail-cuff blood pressure (BP) monitoring showed that ISO/PE group could effectively raise BP starting on the third day (detailed data shown in Figure 1B). At the early stage, we also found a significant up-regulation of heart rate in ISO and ISO/PE groups due to the activation of β1-AR. However, the effect on heart rate is not as pronounced as blood pressure at the end of the second week (ISO/PE: 670.33 ± 41.63 vs. ISO: 664 ± 35.51 vs. Con: 617 ± 40.37 bpm; *P* = 0.0647) (Figure 1C), due to the desensitization of β1-AR under long term ISO stimulation. Interestingly, we did not find a difference in terms of HW/TL ratio between the ISO and ISO/PE groups in the first week, instead, the ISO group displayed a slight rise compared to ISO/PE group. That result was the same as Pan and Matthias report [29]. With the development of HF, the HW/TL ratio was significantly higher in the ISO/PE group than the ISO group (ISO/PE: 11.4 ± 1.03 vs. ISO: 9.07 ± 1.31 vs. Con: 7.76 ± 0.53 mg/mm; *P* = 0.0020) (Figure 1D) at the end of the second week. Also, the decrease of ejection fraction and fraction shortening were more pronounced in the ISO/PE group compared to the ISO group at the end of the second week (EF: ISO/PE: 44.57 ± 5.35% vs. ISO: 45.2 ± 6.88 vs. Con:70.32 ± 5.57, *P* < 0.0001) (Figures 2B–E). The detailed data of ultrasound results are shown in Table 2. It was evident from the results that although both modeling methods were effective in inducing a decrease in cardiac function in mice 2 weeks after the modeling, the ISO/PE group displayed a pronounced HF phenotype compared to the ISO group.

**Table 2:** Echocardiographic measurements.

Times	First week				Second week			
Group	Con	ISO	ISO/PE	*P*	Con	ISO	**ISO/PE**	***P***
SV(mm3)	0.1 ± 0.02	0.09 ± 0.02	0.08 ± 0.04	0.3057	0.1 ± 0.02	0.08 ± 0.03	0.07 ± 0.03	0.4653
LVESV(ml)	0.04 ± 0.02	0.05 ± 0.02	0.06 ± 0.03	0.5330	0.05 ± 0.03	0.13 ± 0.05	0.1 ± 0.04	0.1344
LVEDV(ml)	0.1 ± 0.04	0.14 ± 0.03	0.14 ± 0.07	0.4932	0.15 ± 0.05	0.21 ± 0.08	0.17 ± 0.07	0.5437
IVS(s)	0.27 ± 0.07	0.36 ± 0.05	0.38 ± 0.02	0.0764	0.4 ± 0.02	0.44 ± 0.05	0.39 ± 0.04	0.2729
IVS(d)	0.23 ± 0	0.2 ± 0.02	0.24 ± 0.06	0.4640	0.26 ± 0.05	0.31 ± 0.04	0.31 ± 0.04	0.2774
LVPW(s)	0.32 ± 0.05	0.48 ± 0.02	0.44 ± 0.02	0.0021	0.47 ± 0.06	0.48 ± 0.04	0.41 ± 0.11	0.5838
LVPW(d)	0.26 ± 0.02	0.31 ± 0.04	0.28 ± 0.06	0.3944	0.39 ± 0.07	0.38 ± 0.06	0.35 ± 0.07	0.7651

Summary of 1-week and 2-week cardiac ultrasound data.

Abbreviations: SV, stroke volume; LVESV, left ventricular end-systolic volume; LVEDV, left ventricular end-diastolic volume; IVS (s), interventricular septal diameter at end systole; IVS(d), interventricular septal diameter at end-diastole; LVPW(s), left ventricular posterior wall thickness at end-systole; LVPW(d), left ventricular posterior wall thickness at end-diastole. Data were presented as mean ± SD. *P*-values were presented as numerical values.

### 3.2 ISO/PE group displayed both activations of RAAS and SNS

The SNS and RAAS are two interdependent systems. β1-adrenergic receptor stimulation of the adrenal glands induces renin secretion and hence leads to the production of the vasoconstrictor Ang-II, while in turn, Ang-II stimulates sympathetic nerve ends enhancing the release of catecholamines (25). In addition, ISO is promoting vasodilatation and a reduction in blood pressure via β2-AR activation on smooth muscle cells (26), while PE acts as an α1-AR agonist and hence a vasoconstrictor. We, therefore, hypothesized that the stabilization in BP is based on RAAS activation and hence increased Ang-II levels.

**Figure 3:**
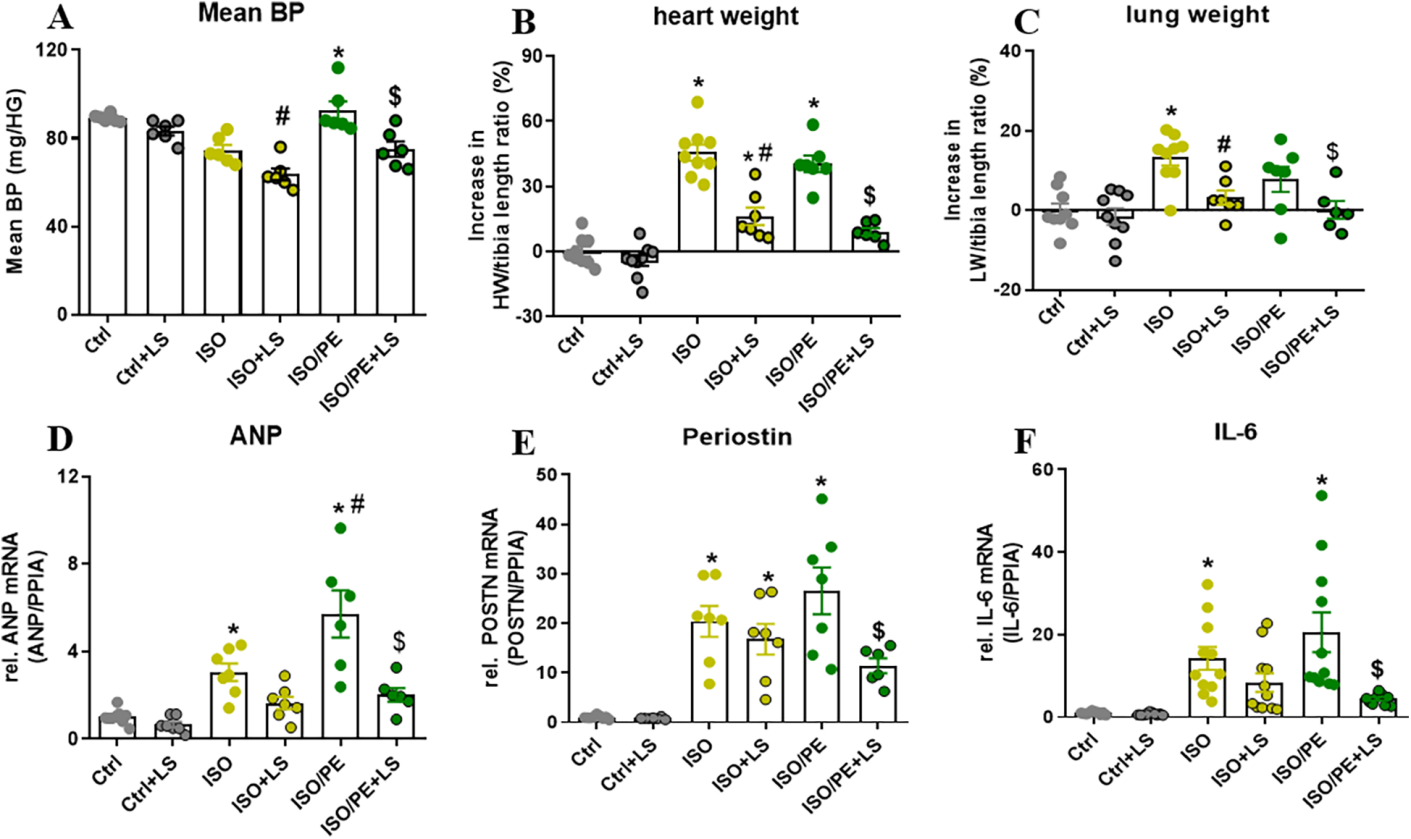
Contribution of RAAS activation in ISO/PE induced hypertrophic growth. **(A)** Mean BP measured by tail-cuff 24 h after minipump implantation and 48 h our losartan was added to the drinking water. **(B)** Percent increase in heart weight/tibia length (HW/TL) ratios renormalized to naCl. **(C)** Percent increase in lung weight/tibia length (LW/TL) ratios renormalized to NaCl. **(D–F)** Gene regulation (qPCR, whole heart tissue) in mice subjected to chronic perfusion of either ISO or ISO/PE (30 mg/kg/day). Values are shown as mean ± SEM; one-way-ANOVA, plus Sidak post-test, *p < 0.05 vs. respective controls, #*p* < 0.05 vs. ISO, $*p* < 0.05 vs. ISO/PE.

To analyze the impact of Ang-II on BP, mice receiving catecholamines stimulation were additionally treated with the ATR1 receptor antagonist losartan for 7 days. Blood pressure measurement after 24 h performed by tail-cuff confirmed that BP is stabilizing in the ISO and ISO/PE control groups, although with a significant delay in the ISO group. The average of level blood pressure was attenuated in losartan-treated animals (Figure 3A). Moreover, during this early stage of catecholamine exposure, losartan almost completely abolished hypertrophic growth (Figure 3B). The transcriptional regulation was more prominent affected in the ISO/PE group leading to a significant reduction in the expression of ANP, Periostin, and IL-6 (Figures 3D–F). Taken together, our data confirmed that RAAS has a major impact on the outcome of co-ISO/PE stimulation.

### 3.3 ISO/PE group showed a higher hypertrophic gene expression and collagen deposition compared to the ISO group

**Figure 4:**
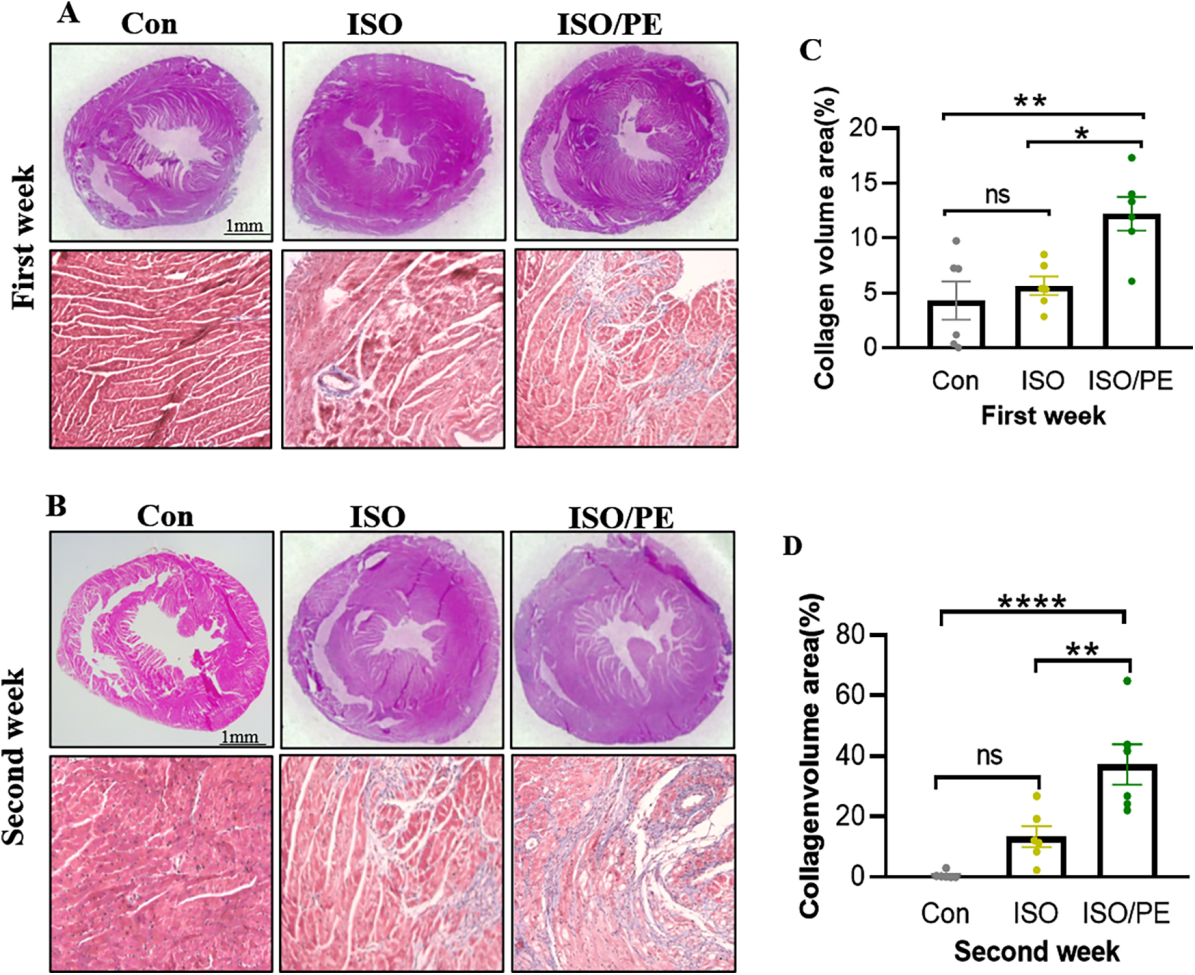
Cardiac hypertrophy growth and collagen deposition. **(A,B)** The mice were drug-molded at 1 and 2 weeks (*n* = 6 in each group), and the hearts were harvested for paraffin-embedded sections and stained with HE and Masson. **(C,D)** Using Image J to quantify the degree of fibrosis in the Masson stain, the ISO/PE group exhibited a significant fibrotic phenotype. Data are presented in the form of mean ± SEM. *P* values were determined by repeated measures one-way ANOVA. *P < 0.05, **P < 0.01, ****P < 0.0001.

**Figure 5:**
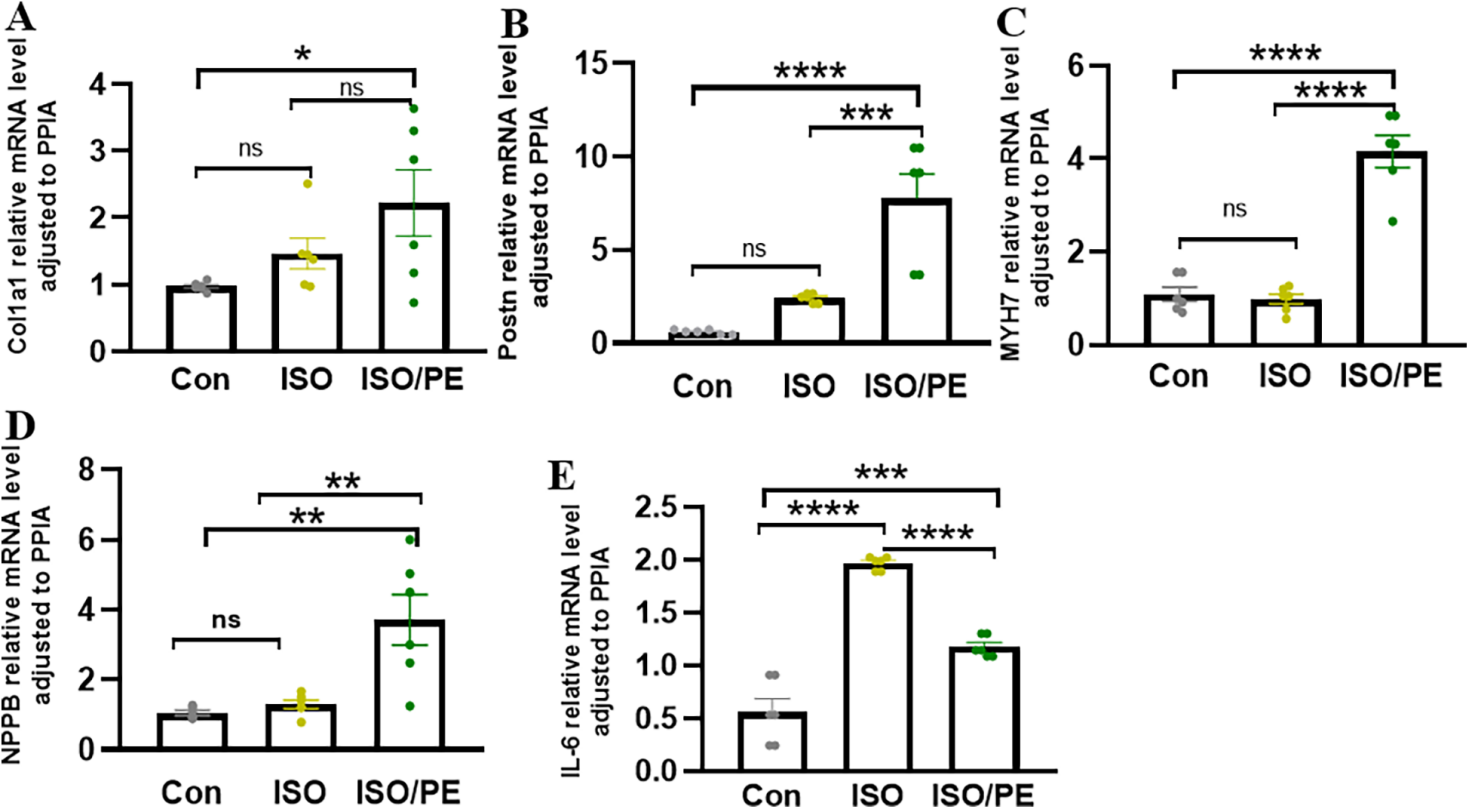
HF associated genes were significantly upregulated in the ISO/PE group. **(A,B)** Two-week post-modeling cardiac tissues were subjected to qPCR (*n* = 6 in each group). Fibrosis-related genes *Postn* and *Col1a1*; **(C,D)** hypertrophy-related gene *Myh7* and heart failure marker gene *Nppb* were clearly upregulated in the ISO/PE group compared with the ISO and control groups. **(E)** The expression of the inflammation-related gene *IL-6* tended to be more significantly upregulated in the ISO group. Data are presented in the form of mean ± SEM. *P* values were determined by repeated measures one-way ANOVA. *P < 0.05, **P < 0.01, ***P < 0.001, ****P < 0.0001.

To observe the degree of cardiac hypertrophy and fibrosis, we executed the mice at one week and two weeks after modeling and took heart tissue for paraffin-embedded sectioning. Then we performed HE as well as Masson staining of cardiac tissues, and the results showed that both ISO and ISO/PE groups could effectively induce ventricular hypertrophy and fibrosis (Figures 4A,B). Quantification results also showed that the fibrotic phenotype of the ISO/PE group was superior to that of the ISO group. As shown in Figure 4C, after one week of dosing, a definite fibrosis phenotype was observed in the ISO/PE group, with the percentage of fibrosis area up to 12.23 ± 3.76%. At this time, the fibrotic phenotype of the ISO group was not yet obvious, and the collagen percentage was 5.66 ± 2.07%. Two weeks after micro-osmotic pump implantation, the fibrosis of the ISO/PE group was more obvious and the percentage of collagen was higher than ISO group (ISO/PE: 37.27 ± 16.32% vs. ISO: 13.39 ± 8.56% vs. Con:0.64 ± 1.14%, *P* < 0.0001) (Figure 4D). This was further validated by the results of the subsequent RT-qPCR (Figure 5). There was a clear upregulation of fibrosis-related genes such as Col1a1 and Postn in the ISO/PE group. AS for Col1a1, ISO/PE group has more than a 2-fold elevation compared to the control group and a 1.5-fold increase compared to the ISO group (*P* = 0.0405). Postn appeared more clearly upregulated in the ISO/PE group, with more than a 12-fold increase compared to the control group; also more than a 3-fold increase compared to the ISO group (*P* < 0.0001) (Figure 5A). In addition, the hypertrophy gene Myh7 was also more obviously up-regulated in the ISO/PE group (more than a 3-fold increase compare to the control group and more than a 4-fold increase compare to the ISO group) (*P* < 0.0001) (Figure 5B). The heart failure marker gene Nppb was also more significantly overexpressed in ISO/PE group (more than a 3-fold increase compare to the control group and more than a 2-fold increase compare to the ISO group) (*P* = 0.0008) (Figure 5C). However, we also found that the upregulation of the inflammation-related gene IL-6 was significantly higher in the ISO group than in the ISO/PE group (more than a 3-fold increase compared to the control group and has onefold increase compared to the ISO/PE group) (*P* < 0.0001) (Figure 5D). Taken together, our experimental data further demonstrate that the co-administrated ISO and PE-induced mouse model displayed a higher hypertrophic gene expression and collagen deposition.

### 3.4 α1-AR activation contributed to more hypertrophic growth through higher phosphorylation of ERK1/2

**Figure 6:**
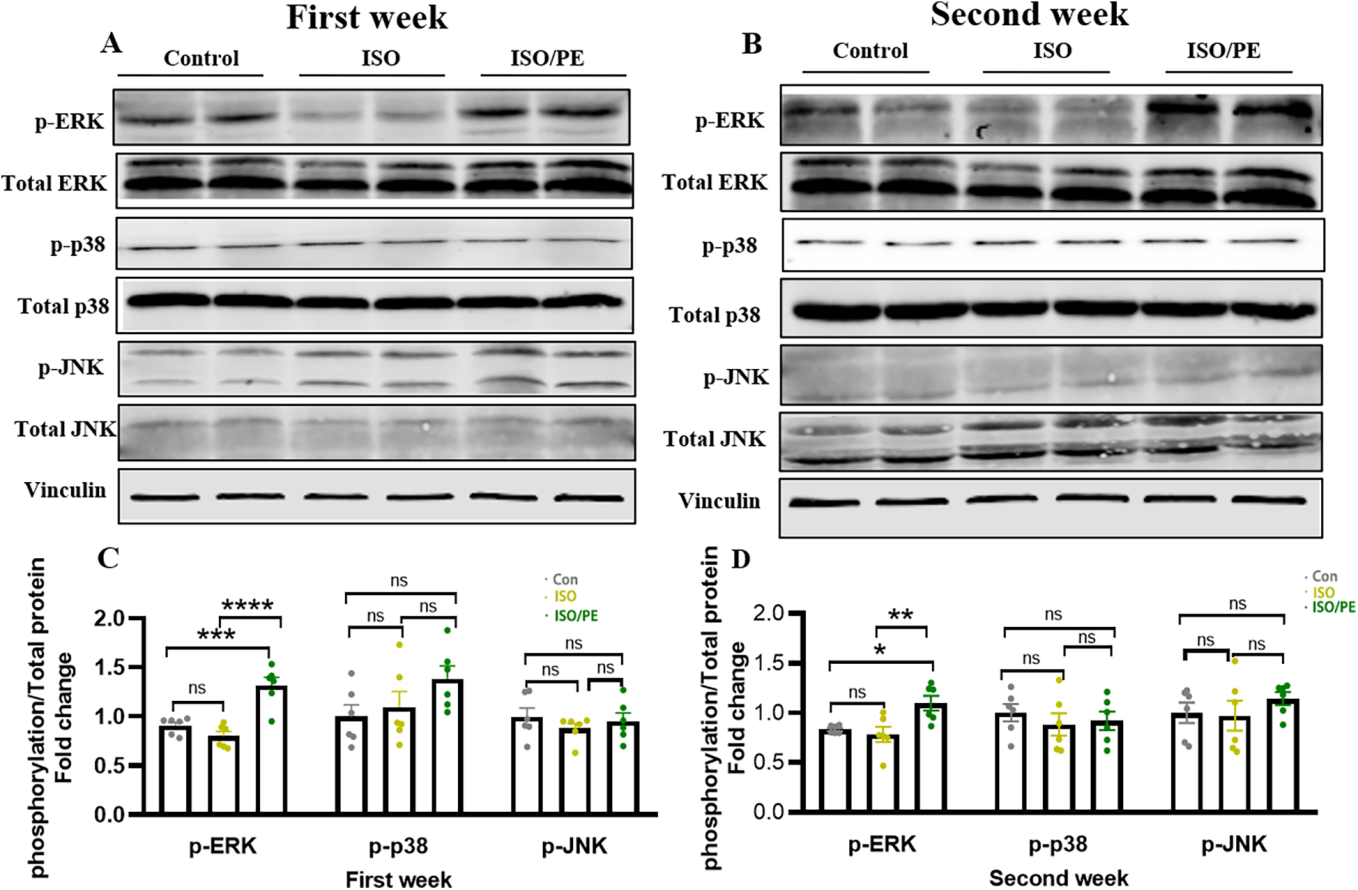
ISO/PE group showed α 1-activation induced phosphorylation of ERK1/2. **(A–D)** The phosphorylation and total protein levels of ERK, JNK and p38, the major protein components of the MAPKs family, were measured after lysis of the tissues using lysis solutions with protease inhibitors and phosphatase inhibitors in the 1-week and 2-week modeling groups (*n* = 6 in each group), respectively. Vinculin was used as an internal reference. Data are presented in the form of mean ± SEM. *P* values were determined by repeated measures one-way ANOVA. *P < 0.05, **P < 0.01, ***P < 0.001, ****P < 0.0001.

Mitogen-activated protein kinase (MAPK) signaling pathway plays a key role in the functional regulation of eukaryotic cells (27). The MAPK pathway is involved in cellular inflammatory responses, cell survival, stress responses, and tumor growth (28), and includes three major executive proteins: extracellular signal-regulated kinase (ERK), c-Jun NH2-terminal kinase (JNK), and p38 (29). Previous studies have confirmed that the upregulation of ERK1/2 phosphorylation played a key role in cardiac hypertrophy and fibrosis (30). And as early as 2014, it was shown that α1-ARs can contribute to the development of cardiac hypertrophy through ERK1/2 (31). Thus, we detected the phosphorylation levels of ERK1/2 and other members of MAPKs. We found that phosphorylation of ERK1/2 was upregulated in the ISO/PE group one week after modeling, a 1.4-fold increase compared to the control group (Figure 6A) and a 1.6-fold increase compared to the ISO group (*P* < 0.0001). Two weeks later, the phosphorylation of ERK1/2 was still significantly upregulated in ISO/PE group, 1.3-fold increase compare to the control group and 1.4-fold increase compared to the ISO group (*P* = 0.0061) (Figure 6B). At the same time, we found that the phosphorylation levels of two other important members of the MAPKs family, JNK and p38, did not show significant differences between the three groups (Figure 6). Thus, our results further confirmed that activation of ERK1/2 under α1-ARs (32) was the main reason for the hypertrophic growth.

### 3.5 A comparison in RNA-seq data among three groups

**Figure 7:**
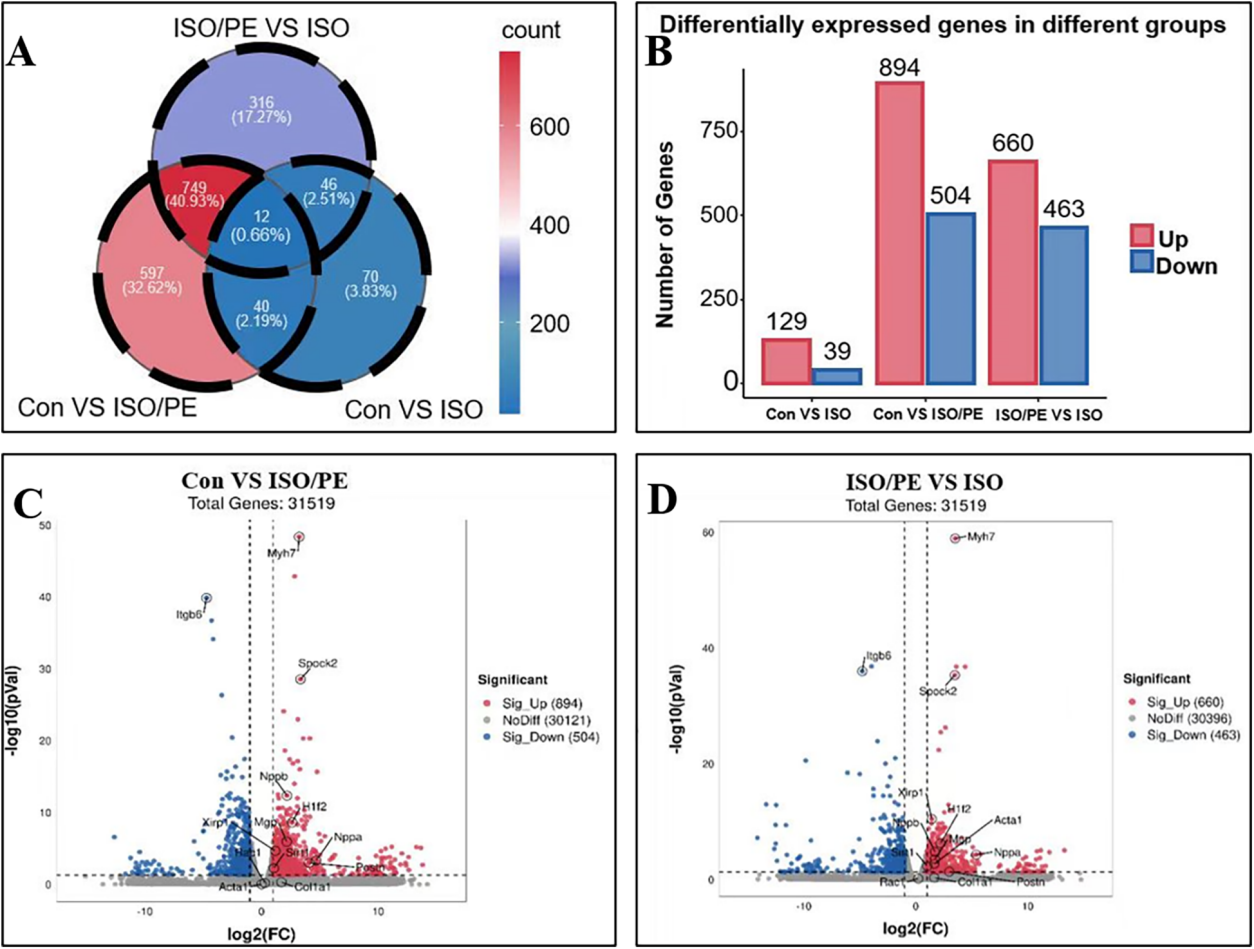
Gene expression changes were more significant in the ISO/PE group. **(A,B)** Myocardial tissues from the control, ISO and ISO/PE groups were sequenced for transcriptomic sequencing (*n* = 3 in Control group, *n* = 4 in ISO group, *n* = 3 in ISO/PE group); a two-by-two comparison between the three groups showed that the ISO/PE group showed the most significant up-regulation and down-regulation of gene expression compared with the control group, followed by the inter-group comparison between the ISO/PE and ISO groups, followed by the difference between the ISO and control groups. **(C,D)** Compared to the control and ISO groups, the hypertrophy gene *Myh7* and the heart failure marker gene *Nppb* were clearly upregulated in the ISO/PE group.

To investigate the gene expression in different treatment groups, we performed RNA sequencing on the left ventricular tissues of mice. The difference genes between the two comparisons of the groups were represented by Venn diagrams. Compared with the control group, the ISO group had 129 up-regulated genes and 39 down-regulated genes, while the ISO/PE group had 894 up-regulated genes and 504 down-regulated genes compared to the control group, both of which were much higher than the ISO group. The ISO/PE group also showed a significant change in gene expression compared to the ISO group, with 660 up-regulated genes and 463 down-regulated genes (Figures 7A,B). Compared with the control group, the heart failure marker genes Nppa and Nppb; fibrosis-related gene Postn, and hypertrophy-related gene Myh7 were upregulated in the ISO/PE group (Figure 7C). Compared with the ISO group, the heart failure marker genes NPPa and NPPb; fibrosis-related gene Postn and hypertrophy-related gene Myh7 also showed clear upregulation in the ISO/PE group (Figure 7D).

**Figure 8:**
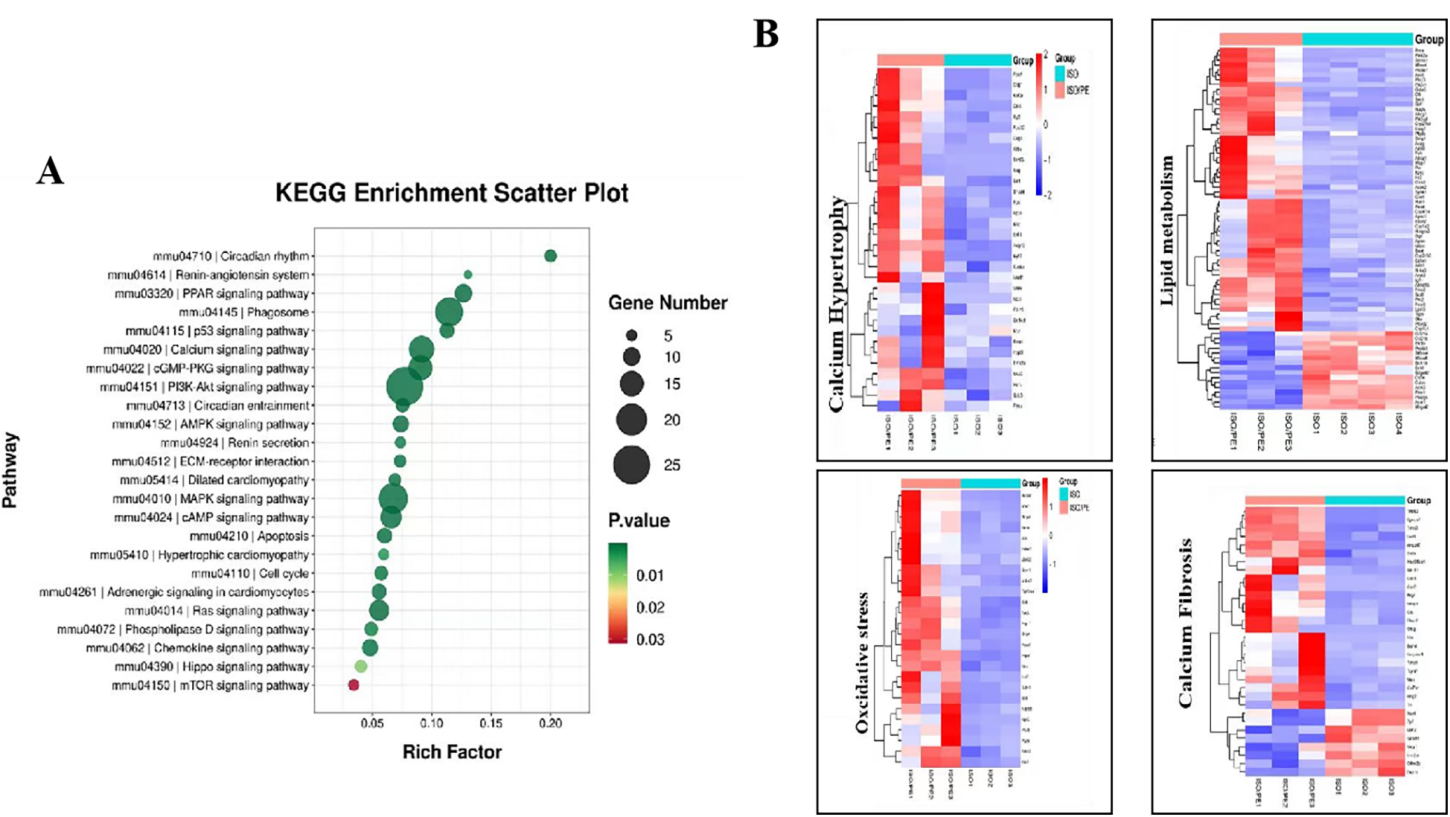
KEGG terms identified by pathway enrichment analysis of the indicated subgroups. **(A)** KEGG gene enrichment analysis of the differential genes among the control, ISO and ISO/PE groups revealed that the differential genes were heavily enriched in RAAS, AMPKs and other pathways. **(B)** Gene list taken from the indicated GO terms summarized as myocardial hypertrophy, fibrosis, oxidative stress and lipid metabolism.

To better understand the functions and action pathways of the above differentially expressed genes, we used KEGG gene enrichment analysis to predict them. Many of the differential genes are components of the RAAS and MAPKs pathways (Figure 8A). The genes upregulated by additional PE supplementary could be clustered into four groups: myocardial hypertrophy, fibrosis, oxidative stress, and lipid metabolism (Figure 8B). In addition to hypertrophy and fibrosis genes, which are also upregulated by conventional modeling methods, the modeling approach used in this experiment can also clearly upregulate genes related to oxidative stress and lipid metabolism pathways, which is of great value in studying the complex factors affecting the development of heart failure.

## 4 Discussion

In this study, we constructed a novel mouse heart failure model by the combination use of ISO/PE. Our modeling approach has clear advantages over traditional drug-induced heart failure models. Morphologically, we confirmed the better and faster induction of heart failure in the ISO/PE group by gross indicators, which were confirmed by cardiac ultrasound monitoring and the ratio of HW/TL. At the histological and molecular levels, we confirmed by HE and Masson staining that the ISO/PE group could better induce cardiac hypertrophy and fibrosis; these results were further validated by qPCR and seq-data, respectively. At the same time, the seq-results showed that the expression of genes related to oxidative stress and fatty acid metabolism could also be upregulated. In addition to the well-known activation of SNS, ISO/PE is also effective in activating RAAS, which has been verified through losartan-blocking Ang-II receptors.

Heart failure has a complex pathogenesis and a high morbidity and mortality rate (33). The SNS, RAAS, and natriuretic peptide system play a key regulatory role in the development of heart failure. Among them, the activation of SNS and RAAS played a facilitating role in the progression of heart failure, and the two systems can influence each other (34). The natriuretic peptide system consists of three bioactive peptides, atrial natriuretic peptide (ANP) and brain (or B-type) are expressed in the heart, both will be elevated in heart failure (35). In the early stages of heart failure, the natriuretic peptide system can play a protective role in maintaining cardiac function by activating the SNS and RAAS, but long-term activation of the RAAS and SNS will lead to a steady decline in cardiac function (36). Hyperactivation of the sympathetic system and RAAS have a crucial effect on cardiac remodeling, and this effect is achieved through a combination activation of α-ARs and β-ARs (37). Therefore, the simultaneous activation of α-AR and β-AR can undoubtedly better simulate the development of heart failure. Previously used ISO-induced heart failure mouse models were mainly achieved through the activation of β-ARs, more prominently by upregulation of inflammatory gene expression (38). By adding the α1-AR agonist PE, our experimental results showed effective activation of the SNS and RAAS. As expected, the final experimental results confirm that ISO/PE modeling is indeed better than conventional ISO modeling.

Activation of α1-AR by various agonists induces a hypertrophic response characterised by immediate activation of early genes (c-Fos, c-Jun) and reactivation of “fetal” genes [c-myc, atrial natriuretic peptide (ANP), α-skeletal actin and β-myosin heavy chain] (22). Similarly, animals develop cardiac hypertrophy after prolonged infusion of low-dose NE or PE. β-ARs have been reported in relation to HF inflammation. Previous studies have shown that β-ARs are involved in the inflammatory response of cardiomyocytes and immune cells (39). The sympathetic nervous system is considered to be an important regulator of the inflammatory response. xiao et al. demonstrated that β1-AR mediated ISO-induced activation of inflammatory vesicles and inflammatory responses in cardiomyocytes. The activation of β-ARs resulted in elevated levels of cAMP in the myocardium (21). cAMP is a known mediator of anti-inflammatory responses, and cAMP-dependent signalling has been pharmacologically used to treat inflammatory diseases. cAMP can act as a positive regulator of a variety of inflammatory genes. In ISO-induced HF, cardiac overexpression of phosphodiesterase 4B, a cAMP-hydrolysing protein, attenuates the β-AR response and maladaptive remodelling, suggesting a pathogenic role for cAMP in sympathoexcitation-associated HF (40).

In the 1980s and 1990s, the MAPKs family, as members of the protein kinase family, received attention from researchers for their involvement in cell cycle regulation and transduction of various signaling pathways (41). ERK, as the first recognized member of the mammalian MAPKs family, mainly includes two types of ERK1 and ERK2, with 85% similarity, and both are widely expressed in various types of cells, including cardiomyocytes (42). The effective induction of ERK1/2 phosphorylation by α1-AR agonism has been demonstrated in previous cellular experiments, which is also consistent with our experimental results (43). Previous studies also found that the ERK/MAPK pathway is closely associated with myocardial hypertrophy and fibrotic phenotype (44), which is also consistent with our experimental results. In addition, blocking the ERK signaling pathway can effectively inhibit collagen deposition and apoptosis (45), so the ERK/MAPKs pathway is likely to be a major mediator of heart failure through cardiac remodeling. However, in the development of heart failure, not only the structural changes of the myocardial cells but also the functional changes of the myocardium itself play a crucial role in the process of heart failure, the most prominent of these is the change in energy metabolism (46).

The heart, being a high-energy consumption organ, normally uses fatty acids (especially long-chain fatty acids) as the main energy substrate (47). However, during the heart failure phase, the energy supply ratio of fatty acids decreases, accompanied by significant changes in other energy substrates (46). In 2021, the sodium-glucose cotransporter 2 inhibitor drugs were included in heart failure prevention and treatment guidelines (44). So it has become indisputable that alterations in metabolism during the heart failure phase can serve as an intervention target. Oxidative stress refers to a state of imbalance in the production and processing of reactive oxygen species, which plays a key role in the development of heart failure (48). Since oxidative phosphorylation of mitochondria is the main source of cellular reactive oxygen species, changes in mitochondrial ROS production brought about by alterations in energy metabolism and subsequent changes in mitochondrial function also contribute significantly to the development of heart failure (49). Our study presents a novel approach to heart failure induction in mice, which not only upregulates hypertrophy and fibrosis-related genes but also shows clear upregulation of oxidative stress and lipid metabolism pathways. Since the development of heart failure is mediated by a combination of factors, the modeling approach proposed in this study appears to be more consistent with the real-world progression of heart failure.

As a drug-induced heart failure model, our modeling approach not only has the advantage of saving time and effort in traditional drug-induced models but also can activate both SNS and RAAS, which can better simulate the development of heart failure compared to traditional mouse models. Although this modeling approach takes into account the relative complexity of heart failure progression mechanisms, it also has some disadvantages. First, the mice used in this experiment were all males and there were only 6 mice in each group, so the number was relatively small. Second, although we confirmed that the ISO/PE group could activate SNS and RAAS well, the degree of activation of RAAS was not measured. Although this study confirms that the use of ISO/PE is effective in activating multiple pro-heart failure factors, the exact mechanism has not been elucidated. Despite the above shortcomings, it still has good prospects for future basic research as a new method for induction of heart failure models.

## 5 Conclusion

Currently available modeling methods have their own merits and drawbacks, and different research directions require different animal models to meet the research needs. In this experiment, the traditional modeling method can be improved to better simulate the development of heart failure, which is a good modeling solution. Our modeling approach has very clear advantages over conventional modeling. First, it is more reproducible than TAC and anterior descending coronary artery ligation. Second, our approach is faster and more stable than ISO group. More certainly, it is more compatible with the human heart failure process than doxorubicin dosing. In addition, it also activates the RAAS besides the SNS, which more closely resembles the pathological features of the heart failure state than the angiotensin-induced heart failure model alone.

## Data Availability

The original contributions presented in the study are publicly available. This data can be found here: https://www.ncbi.nlm.nih.gov/sra/PRJNA1000310.
